# Sentinel surveillance for influenza A viruses in Lahore District Pakistan in flu season 2015–2016

**DOI:** 10.1186/s12879-021-07021-7

**Published:** 2022-01-06

**Authors:** Saima Hasan, Richard J. Webby, Muhammad Iqbal, Hamad Bin Rashid, Mansur-ud-Din Ahmad, Jawad Nazir, Jennifer DeBeauchamp, Shakera Sadiq, Mamoona Chaudhry

**Affiliations:** 1 Department of Epidemiology and Public Health, University of Veterinary and Animal Sciences, Lahore, Pakistan; 2 World Health Organization Collaborating Center for Studies on the Ecology of Influenza in Animals and Birds, Department of Infectious Diseases, St. Jude Children’s Research Hospital, Memphis, TN USA; 3 Department of Surgery and Pet Sciences, University of Veterinary and Animal Sciences, Lahore, Pakistan; 4 Department of Pathobiology, Riphah Veterinary College, Riphah International University, Lahore, Pakistan; 5 Department of Microbiology, University of Veterinary and Animal Sciences, Lahore, Pakistan; 6 Virology Laboratory, Treidlia Biovet, Seven Hills, Blacktown, NSW Australia

**Keywords:** Influenza, Active surveillance, Influenza like illness (ILI), Severe acute respiratory illness (SARI), Pandemic, Seasonal influenza

## Abstract

**Background:**

Influenza A virus (IAV) remains an important global public health threat with limited epidemiological information available from low-and-middle-income countries. The major objective of this study was to describe the proportions, temporal and spatial distribution, and demographic and clinical characteristics of IAV positive patients with influenza like illness (ILI) and severe acute respiratory illness (SARI) in Lahore, Pakistan.

**Methods:**

Prospective surveillance was established in a sentinel hospital from October 2015 to May 2016. All eligible outpatients and inpatients with ILI or SARI were enrolled in the study. Nasal and/or throat swabs were collected along with clinico-epidemiological data. Samples were tested by real-time RT-PCR (rRT-PCR) to identify IAV and subtype. The descriptive analysis of data was done in R software.

**Results:**

Out of 311 enrolled patients, 284 (91.3%) were ILI and 27 (8.7%) were SARI cases. A distinct peak of ILI and SARI activity was observed in February. Fifty individuals (16%) were positive for IAV with peak positivity observed in December. Of 50 IAV, 15 were seasonal H3N2, 14 were H1N1pdm09 and 21 were unable to be typed. The majority of IAV positive cases (98%) presented with current or history of fever, 88% reported cough and 82% reported sore throat. The most common comorbidities in IAV positive cases were hepatitis C (4%), obesity (4%) and tuberculosis (6%). The highest incidence of patients reporting to the hospital was seen three days post symptoms onset (66/311) with 14 of these (14/66) positive for IAV.

**Conclusion:**

Distinct trends of ILI, SARI and IAV positive cases were observed which can be used to inform public health interventions (vaccinations, hand and respiratory hygiene) at appropriate times among high-risk groups. We suggest sampling from both ILI and SARI patients in routine surveillance as recommended by WHO.

**Supplementary Information:**

The online version contains supplementary material available at 10.1186/s12879-021-07021-7.

## Background

Influenza is a highly contagious pathogen causing a significant burden of morbidity and mortality worldwide [1]. Diverse seasonal trends across geographical regions are observed with annual epidemics and periodic pandemics caused by the emergence of novel IAVs to which humans have limited pre-existing immunity [2].

In temperate regions of the world, seasonal winter influenza epidemics increase overall morbidity and mortality and cause significant economic losses due to absenteeism from work [3, 4]. However, comparatively limited amounts of data are available concerning the burden of influenza in tropical and subtropical countries [5].

Understanding influenza seasonality and regional variation is important for developing timely preventive measures including influenza vaccination and other control strategies. In 2012, the WHO reported that 84% of countries in tropical and subtropical regions have a single peak of influenza activity which usually extends from November–December through February–March with only 4.2% having year round activity [6, 7]. Considering resource limitations, sentinel surveillance has been found superior to laboratory-based or population-based surveillance to estimate the viral determinants of ILI in developing countries.

According to World Bank, Pakistan is a lower-middle-income country (https://data.worldbank.org/country/pakistan) with a population of ~ 220 million [8] and a weak healthcare delivery system [9]. The per capita annual household income in Pakistan was US$650.64 in 2016. The climate of the country is tropical to temperate with scattered arid areas in the southern regions [10]. Influenza viruses have always been considered a significant threat to global public health [11]. Since 2009 A (H1N1pdm09), seasonal influenza (H3N2) and influenza B viruses have circulated in humans [12]. Limited data are available for circulation patterns of these viruses in Pakistan although epidemiological and clinical factors associated with H1N1pdm09 infection during the 2009–2010 pandemic [13, 14] and zoonotic influenza [15–17] have been reported. A recent study reported the positivity rate, seasonality, and epidemiological and clinical characteristics of ILI and SARI patients at eight sentinel sites in Pakistan during 2008–2017 [18]. In current study, one sentinel site in Lahore, which is the second most populous city of Pakistan with a population > 11 million, was included [19].

In the current study we focused on a major tertiary-care hospital in Lahore with high patient loads as the sentinel site to enroll patients using syndromic definitions for respiratory infections. We sought to describe the proportions, temporal and spatial distribution, and demographic and clinical characteristics of IAV positive ILI and SARI cases and to estimate the proportion of seasonal H3N2 and H1N1pdm09 subtypes among positive IAV cases.

## Methods

### Study site

**Fig. 1:**
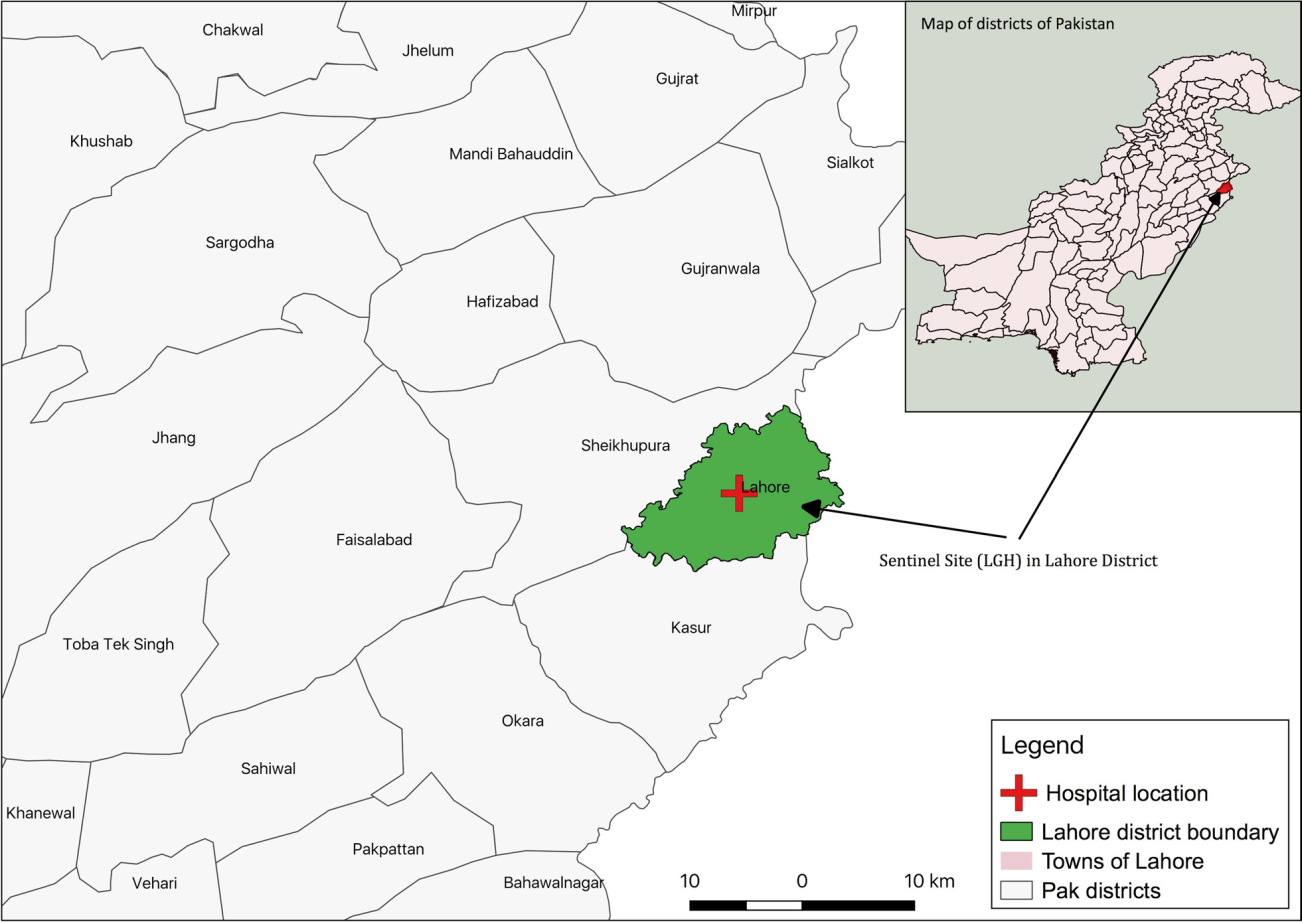
Sentinel site (LGH) in Lahore District

Lahore is the capital city of the Punjab province of Pakistan (31°32′59″N 74°20′37″E) with a population of 11,119,985 [19]. The district is bounded on the north and west by the Sheikhupura District, on the south by the Kasur District and on the east by the Indian border (Fig. 1). Lahore has a semi-arid climate and experiences four distinct seasons i.e. summer, winter, autumn and spring [20] with June being the hottest month and January the coolest characterized by intense fog and smog, especially over the past 2–3 years [21].

The Lahore General Hospital (LGH) is a major public sector hospital and referral center for the population of Lahore as well as neighboring districts and includes emergency and outpatient clinics and is affiliated with Post Graduate Medical Institute (PGMI) Lahore. The capacity of LGH is 1600 beds. Approximately 14,00,000 outpatients visit various outdoor clinics of the hospital e.g. Medical unit I, II, III, Pediatrics, Neurology, Hepatic, Urology, Orthopedic, ENT, Gynecology etc. annually while the number of outpatient visits is 4000–4500 daily. The number of patients visiting emergency department is 2500–3000 daily. The hospital has 14 ventilators, 10 ICU beds, and 20 isolation beds (https://lgh.punjab.gov.pk/clinical_services).

### Study population

We prospectively enrolled outpatients with influenza like illness (ILI) and inpatients with severe acute respiratory infection (SARI) according to WHO definition (see below) from those visiting a designated seasonal influenza desk for symptom screening (located within the LGH outpatient department) from Monday to Saturday during official working hours from October 2015 to May 2016. Due to logistic and access issues, we selected one sentinel hospital site from 14 Tertiary Care hospitals and sampled 5–6 patients visiting the hospital on each day, who fulfilled the ILI and/or SARI definitions. These 5–6 patients were selected conveniently from those patients, who visited the hospital in the early hours. Eligible patients were provided with an explanation of the study rationale and sample collection procedures and invited to participate voluntarily. The Independent Ethics Committee (IEC) Bioequivalence Study (Be St) Center, University of Veterinary and Animal Sciences, Lahore (Letter no. IEC-438) approved study protocol.

The standardized and January 2014-revised WHO ILI and SARI case definitions were used. A case of ILI was defined as an outpatient with history of fever (measured temperature ≥ 38 °C) during the past 10 days, and cough without the need of hospitalization. The SARI cases were defined as patients with an acute respiratory illness with onset during the previous 10 days requiring at least an overnight hospitalization and having history of fever or measured temperature of ≥ 38 °C, and cough [22]. Those who did not fulfill the case definition criteria or refused to participate were excluded from the study.

The geographical locations (street addresses) of enrolled patients were located on Google maps. A dot map of distribution of positive and negative cases of influenza A was produced using QGIS software version 3.2 (Open Source Geospatial Foundation Project, Boston, MA, USA, available at http://qgis.osgeo.org).

### Data collection

Using a pretested questionnaire (Additional file 1), epidemiological data about socio-demographics, co-morbidities, exposure status, vaccination, and travel history was gathered from enrolled patients in a face-to-face interview in the local language. A unique coded identification number was given to each questionnaire and specimen tube from the same patients to keep the data confidential.

### Sample collection and laboratory analysis

A registered clinical officer collected nasopharyngeal or oropharyngeal swabs from willing participants [23]. The specimen was immediately transferred into a cryovial tube containing 2-3 ml of viral transport medium. All collected specimens were transported at 4 °C to the Disease Surveillance Laboratory, Department of Epidemiology & Public Health, University of Veterinary and Animal Sciences, Lahore, Pakistan. On arrival in the laboratory, all specimens were divided into small aliquots in a biosafety level-II cabinet (BSL-2) and were stored at or below − 70 °C before shipment to the WHO Collaborating Center for Studies on the Ecology of Influenza, St. Jude Children's Research Hospital, Memphis, Tennessee, USA for influenza testing [23, 24] by using real-time RT-PCR (rRT-PCR) protocol as described by the US Centers for Disease Control and Prevention [25] for IAV. Positive IAV specimens were further subtyped for seasonal H3N2 and H1N1pdm09 by rRT-PCR using specific primers, probes [26].

### Statistical analysis

Questionnaire data were entered into Epidata version 3.1 (available at http://www.epidata.dk) and validated for errors and inconsistencies. We used R software version 3.2.3 [27] for analysis of socio-demographic and clinical characteristics. Graphs were generated to present spatio-temporal trends of IAV and subtypes (seasonal H3N2 and H1N1pdm09). The denominator for calculation of various proportions was the total number of cases (all diseased) who visited the hospital during study period (n = 513,126) and total number of ILI and SARI patient enrolled in study (n = 311).

## Results

### Characteristics of ILI and SARI cases

From October 2015 to May 2016, a total of 513,126 patients reported to the LGH surveillance desk designated for infectious diseases. A total of 4568 patients visited the designated seasonal influenza desk with ILI or SARI symptoms. Of the 4568 patients, 311 were invited to participate and were enrolled in study. Among 311, 284 were ILI (91.3%) and 27 were SARI (8.7%) patients. The mean age among all enrolled patients was 29.20 years (median 27 years; range: 0.33–80 years). Most ILI (70.1%, mean = 29 years) and SARI patients (66.67%, mean = 29 years) were under the age of 30 years. The majority of cases were females (n = 202, 65%). Among these 202, 184 were ILI patients and 18 were SARI cases. Out of these 202 females, 140 were married and among these, 91.4% (128/140) were ILI patients and 8.6% (12/140) were SARI patients. Among married ILI female patients (n = 128), 3 (2.3%) were in first trimester of pregnancy, and 4 (3.1%) were in second trimester of pregnancy; none were in the third trimester. About 94.5% (n = 121) female ILI patients were married but not pregnant. Among married SARI female patients (n = 12), 1 (8.3%) was in the second trimester and 1 (8.3%) was in the third trimester. The number of ILI and SARI patients was higher among housewife’s compared to other occupations. In overall, a single-family system was more common (187/311: 60.1%) and families had an average monthly income of US$172. No deaths were recorded among enrolled cases. About 31.5% of patients (98/311) reported that they self-medicated with antibiotics (available over the counter in Pakistan). Among ILI patients, 27% reported self-medication with antibiotics and among SARI, 29.6% reported use of antibiotics. No patient reported the use of any antiviral drug. Out of 311 patients, 99.4% (n = 304) were unaware of the availability of any influenza vaccine. Only two patients (0.6%) knew about the seasonal influenza vaccine and one (0.3%) was vaccinated against influenza and they were among ILI cases. Among ILI patients, 16 (5.6%) were vaccinated with pneumococcal vaccine, while in SARI cases, one (3.7%) was vaccinated with pneumococcal vaccine. Majority of ILI (80.9%) and SARI (88.8%) had no travel history. Similarly, majority of ILI (87.6%) and SARI (88.8%) reported no contact with poultry. Most of the ILI (94.3%) and SARI (92.5%) did not used public transport (Tables 1 and 2).

**Table 1:** Distribution of various exposure characteristics among IAV positive ILI patients

Characteristics	ILI cases					
	IAV positive cases (n = 40)			IAV Positive cases (n = 40)	IAV negative cases (n = 244)	Total (n = 284)
	H1N1pm09	H3N2	Untyped			
Age						
0–15	0 (0.0%)	1 (2.5%)	2 (5.0%)	3 (5.5%)	36 (14.7%)	39 (13.7%)
16–30	6 (15.0%)	8 (20.0%)	8 (20.0%)	22 (55.0%)	105 (43.0%)	127 (44.7%)
31–45	1 (2.5%)	3 (7.5%)	5 (12.5%)	9 (22.5%)	66 (27.0%)	75 (26.4%)
46–60	2 (5.0%)	1 (2.5%)	3 (7.5%)	6 (15.0%)	31 (12.7%)	37 (13.0%)
> 60	0 (0.0%)	0 (0.0%)	0 (0.0%)	0 (0.0%)	6 (2.4%)	6 (2.11%)
Gender						
Female	4 (10.0%)	6 (15.0%)	8 (20.0%)	18 (45.0%)	166 (68.0%)	184 (64.7%)
Male	5 (12.5%)	7 (17.5%)	10 (25.0%)	22 (55.0%)	78 (32.0%)	100 (35.2%)
Marital status						
Married	6 (15%)	6 (15%)	12 (30%)	24 (60.0%)	146 (59.8)	170 (59.9%)
Unmarried	3 (7.5%)	7 (17.5%)	5 (12.5%)	15 (37.5%)	97 (39.7%)	112 (39.4%)
Widow	0 (0.0%)	0 (0.0%)	1 (2.5%)	1 (2.5%)	0 (0.0%)	1 (0.4%)
Divorced	0 (0.0%)	0 (0.0%)	0 (0.0%)	0 (0.0%)	1 (2.5%)	1 (0.4%)
Family system						
Single	6 (15.0%)	9 (22.5%)	11 (27.5%)	25 (65.0%)	144 (59.0%)	170 (59.8%)
Extended	3 (7.5%)	4 (10.0%)	7 (17.5%)	14 (35.0%)	100 (41.0%)	114 (40.1%)
Education						
Illiterate	0 (0.0%)	0 (0.0%)	0 (0.0%)	0 (0.0%)	18 (7.4%)	18 (6.3%)
Primary	3 (7.5%)	2 (5.0%)	5 (12.5%)	10 (25.0%)	73 (29.9%)	83 (29.2%)
Secondary	1 (2.5%)	2 (5.0%)	4 (10.0%)	7 (17.5%)	48 (19.7%)	55 (19.3%)
Intermediate	2 (5.0%)	7 (17.5%)	7 (17.5%)	16 (40.0%)	79 (32.4%)	95 (33.4%)
Graduate/post graduate	3 (7.5%)	2 (5.0%)	2 (5.0%)	7 (17.5%)	26 (10.6%)	33 (11.6%)
Income/month in rupees (US$)						
Less than 10,000 (< 96)	1 (2.5%)	2 (5.0%)	1 (2.5%)	4 (10.0%)	50 (20.5%)	54 (19.0)
10,000 to 15,000 (96–144)	3 (7.5%)	9 (22.5%)	12 (30.0%)	24 (60.0%)	133 (54.5%)	157 (55.2%)
15,000 to 20,000 (144–192)	2 (5.0%)	1 (2.5%)	5 (12.5%)	8 (20.0%)	39 (16.0%)	47 (16.5%)
More than 20,000 (> 192$)	3 (7.5%)	1 (2.5%)	0 (0.0%)	4 (10.0%)	22 (9.0%)	26 (9.1%)
Occupation						
Government/private employees	0 (0.0%)	0 (0.0%)	2 (5.0%)	2 (5.0%)	17 (7.0%)	19 (6.6%)
Jobless skilled worker	4 (10.0%)	5 (12.5%)	4 (10.0%)	13 (32.5%)	56 (23.0%)	69 (24.2%)
Housewife	3 (7.5%)	3 (7.5%)	4 (10.0%)	10 (25.0%)	104 (42.6%)	114 (40.1%)
Health professional	0 (0.0%)	0 (0.0%)	0 (0.0%)	0 (0.0%)	2 (0.8%)	2 (0.7%)
Driver	1 (2.5%)	1 (2.5%)	0 (0.0%)	2 (5.0%)	3 (1.2%)	5 (1.7%)
Others	1 (2.5%)	4 (10.0%)	8 (20.0%)	13 (32.5%)	64 (26.2%)	77 (27.1%)
Travel history						
No	7 (17.5%)	11 (27.5%)	13 (32.5%)	31 (77.5%)	199 (81.55%)	230 (80.9%)
Yes	2 (5.0%)	2 (5.0%)	5 (12.5%)	9 (22.5%)	45 (18.4%)	54 (19.0%)
Contact with poultry						
No	9 (22.5%)	11 (27.5%)	14 (35.0%)	34 (85.0%)	215 (88.1%)	249 (87.6%)
Yes	0 (0.0%)	2 (5.0%)	4 (10.0%)	6 (15.0%)	29 (11.9%)	35 (12.3%)
Use of public transport						
No	8 (20.0%)	13 (32.5%)	16 (40.0%)	37 (92.5%)	231 (94.7%)	268 (94.3%)
Yes	1 (2.5%)	0 (0.0%)	2 (5.0%)	3 (7.5%)	13 (5.3%)	16 (5.6%)
Pre-existing condition						
No	4 (10.0%)	11 (27.5%)	13 (32.5%)	28 (70.0%)	179 (73.4)	207 (72.9
Yes	5 (12.5)	2 (5.0%)	5 (12.5)	12 (30%)	65 (26.63)	77 (27.1)
Use of antibiotics						
No	2 (5.0%)	7 (17.5%)	8 (20.0%)	17 (42.5%)	174 (71.3%)	191 (67.3%)
Yes	7 (17.5%)	6 (15.0%)	10 (25.0%)	23 (57.5%)	70 (28.7%)	77 (27.1%)
Use of antiviral						
No	9 (22.5%)	13 (32.5%)	18 (45.0%)	40 (100.0%)	244 (100.0%)	284 (100%)
Yes	0 (0.0%)	0 (0.0%)	0 (0.0%)	0 (0.0%)	0 (0.0%)	0 (0.0%)
Knowing about influenza vaccine						
No	9 (22.5%)	13 (32.5%)	18 (45.0%)	40 (100.0%)	242 (99.2%)	282 (99.3%)
Yes	0 (0.0%)	0 (0.0%)	0 (0.0%)	0 (0.0%)	2 (0.8%)	2 (0.7%)
Vaccinated with influenza vaccine						
No	9 (22.5%)	13 (32.5%)	18 (45.0%)	40 (100.0%)	243 (99.6%)	283 (99.6%)
Yes	0 (0.0%)	0 (0.0%)	0 (0.0%)	0 (0.0%)	1 (0.4%)	1 (0.4%)
Vaccinated with pneumococcal vaccine						
No	9 (22.5%)	13 (32.5%)	18 (45.0%)	40 (100.0%)	228 (93.4%)	268 (94.4%)
Yes	0 (0.0%)	0 (0.0%)	0 (0.0%)	0 (0.0%)	16 (6.6%)	16 (5.6%)
Pregnancy						
First trimester	0 (0.0%)	0 (0.0%)	0 (0.0%)	0 (0.0%)	3 (1.2%)	3 (1.1%)
Second trimester	0 (0.0%)	0 (0.0%)	0 (0.0%)	0 (0.0%)	4 (1.6%)	4 (1.4%)
Third trimester	0 (0.0%)	0 (0.0%)	0 (0.0%)	0 (0.0%)	0 (0.0%)	0 (0.0%)
Not pregnant	6 (15.0%)	6 (15.0%)	10 (25.0%)	22 (55.0%)	113 (46.3%)	135 (47.5%)
Not applicable	3 (7.5%)	7 (17.5%)	8 (20.0%)	18 (45.0%)	124 (50.8%)	142 (50.0%)

**Table 2:** Distribution of various exposure characteristics among IAV positive SARI patients

Characteristics	SARI cases (27)					
	IAV positive cases (n = 10)			IAV positive cases (n = 10)	IAV negative cases (n = 17)	Total (n = 27)
	H1N1pm09	H3N2	Untyped			
Age						
0–15	0 (0.0%)	0 (0.0%)	0 (0.0%)	0 (0.0%)	2 (11.7%)	2 (7.4%)
16–30	4 (40.0%)	1 (10.0%)	2 (20.0%)	7 (70.0%)	8 (47.0%)	15 (55.5%)
31–45	0 (0.0%)	1 (10.0%)	1 (10.0%)	2 (20.0%)	5 (29.4%)	7 (25.9%)
46–60	1 (10.0%)	0 (0.0%)	0 (0.0%)	1 (10.0%)	2 (11.8%)	3 (11.11%)
> 60	0 (0.0%)	0 (0.0%)	0 (0.0%)	0 (0.0%)	0 (0.0%)	0 (0.0%)
Gender						
Female	4 (40.0%)	2 (20.0%)	1 (10.0%)	7 (70.0%)	11 (64.7%)	18 (66.6%)
Male	1 (10.0%)	0 (0.0%)	2 (20.0%)	3 (30.0%)	6 (35.3%)	9 (33.3%)
Marital status						
Married	5 (50.0%)	2 (20.0%)	1 (10.0%)	8 (80.0%)	9 (52.9%)	17 (63.0%)
Unmarried	0 (0.0%)	0 (0.0%)	2 (20.0%)	2 (20.0%)	8 (47.0%)	10 (37.0%)
Widow	0 (0.0%)	0 (0.0%)	0 (0.0%)	0 (0.0%)	0 (0.0%)	0 (0.0%)
Divorced	0 (0.0%)	0 (0.0%)	0 (0.0%)	0 (0.0%)	0 (0.0%)	0 (0.0%)
Family system						
Single	2 (20.0%)	0 (0.0%)	3 (30.0%)	5 (50.0%)	12 (70.5%)	17 (62.9%)
Extended	3 (30.0%)	2 (20.0%)	0 (0.0%)	5 (50.0%)	5 (10.4%)	10 (37.0%)
Education						
Illiterate	0 (0.0%)	0 (0.0%)	0 (0.0%)	0 (0.0%)	1 (5.9%)	1 (3.7%)
Primary	3 (30.0%)	1 (10.0%)	0 (0.0%)	4 (40.0%)	4 (23.5%)	8 (29.6%)
Secondary	1 (10.0%)	0 (0.0%)	0 (0.0%)	1 (10.0%)	2 (11.8%)	3 (11.1%)
Intermediate	1 (10.0%)	1 (10.0%)	2 (20.0%)	4 (40.0%)	8 (47.0%)	12 (44.4%)
Graduate/post graduate	0 (0.0%)	0 (0.0%)	1 (10.0%)	1 (10.0%)	2 (11.8%)	3 (11.1%)
Income/month in rupees (US$)						
Less than 10,000 (< 96)	0 (0.0%)	1 (10.0%)	0 (0.0%)	1 (10.0%)	2 (11.8%)	3 (11.1%)
10,000 to 15,000 (96–144)	3 (30.0%)	1 (10.0%)	2 (20.0%)	6 (60.0%)	8 (47.0%)	14 (51.8%)
15,000 to 20,000 (144–192)	2 (20.0%)	0 (0.0%)	1 (10.0%)	3 (30.0%)	5 (29.4%)	8 (29.6%)
More than 20,000 (> 192$)	0 (0.0%)	0 (0.0%)	0 (0.0%)	0 (0.0%)	2 (11.8%)	2 (7.4%)
Occupation						
Government/private employees	0 (0.0%)	0 (0.0%)	0 (0.0%)	0 (0.0%)	2 (11.8%)	2 (7.4%)
Jobless skilled worker	0 (0.0%)	0 (0.0%)	1 (10%)	1 (10%)	3 (17.6%)	4 (14.8%)
Housewife	2 (20%)	1 (10.0%)	1 (10.0%)	4 (40.0%)	6 (35.3%)	10 (37.0%)
Health professional	0 (0.0%)	0 (0.0%)	0 (0.0%)	0 (0.0%)	1 (5.9%)	1 (3.7%)
Driver	0 (0.0%)	0 (0.0%)	0 (0.0%)	0 (0.0%)	1 (5.9%)	1 (3.7%)
Others	3 (30.0%)	1 (10.0%)	1 (10.0%)	5 (50.0%)	4 (23.5%)	9 (33.3%)
Travel history						
No	5 (50.0%)	2 (20.0%)	2 (20.0%)	9 (90.0%)	15 (88.2%)	24 (88.8%)
Yes	0 (0.0%)	0 (0.0%)	1 (10.0%)	1 (10.0%)	2 (11.8%)	3 (11.1%)
Contact with poultry						
No	4 (40.0%)	2 (20.0%)	3 (30.0%)	9 (90.0%)	15 (88.2%)	24 (88.8%)
Yes	1 (10.0%)	0 (0.0%)	0 (0.0%)	1 (10.0%)	2 (11.8%)	3 (11.1%)
Use of public transport						
No	5 (50.0%)	2 (20.0%)	3 (30.0%)	10 (100%)	15 (88.2%)	25 (92.5%)
Yes	0 (0.0%)	0 (0.0%)	0 (0.0%)	0 (0.0%)	2 (11.8%)	2 (7.4%)
Pre-existing condition						
No	5 (50.0%)	0 (0.0%)	3 (30.0%)	8 (80.0%)	11 (64.7%)	19 (70.4%)
Yes	0 (0.0%)	2 (20.0%)	0 (0.0%)	2 (20.0%)	6 (35.3%)	8 (29.6%)
Use of antibiotics						
No	5 (50.0%)	2 (20.0%)	2 (20.0%)	9 (90.0%)	13 (76.5%)	22 (81.5%)
Yes	0 (0.0%)	0 (0.0%)	1 (10.0%)	1 (10.0%)	4 (23.5%)	5 (18.5%)
Use of antiviral						
No	5 (50.0%)	2 (20.0%)	3 (30.0%)	10 (100%)	17 (100%)	27 (100%)
Yes	0 (0.0%)	0 (0.0%)	0 (0.0%)	0 (0.0%)	0 (0.0%)	0 (0.0%)
Knowing about influenza vaccine						
No	5 (50.0%)	2 (20.0%)	3 (30.0%)	10 (100%)	17 (100%)	27 (100%)
Yes	0 (0.0%)	0 (0.0%)	0 (0.0%)	0 (0.0%)	0 (0.0%)	0 (0.0%)
Vaccinated with influenza vaccine						
No	5 (50.0%)	2 (20.0%)	3 (30.0%)	10 (100%)	17 (100%)	27 (100%)
Yes	0 (0.0%)	0 (0.0%)	0 (0.0%)	0 (0.0%)	0 (0.0%)	0 (0.0%)
Vaccinated with pneumococcal vaccine						
No	5 (50.0%)	2 (20.0%)	3 (30.0%)	10 (100%)	16 (94.1%)	26 (96.3%)
Yes	0 (0.0%)	0 (0.0%)	0 (0.0%)	0 (0.0%)	1 (5.9%)	1 (3.7%)
Pregnancy						
First trimester	0 (0.0%)	0 (0.0%)	0 (0.0%)	0 (0.0%)	0 (0.0%)	0 (0.0%)
Second trimester	0 (0.0%)	0 (0.0%)	1 (10.0%)	1 (10.0%)	0 (0.0%)	1 (3.7%)
Third trimester	0 (0.0%)	1 (10.0%)	0 (0.0%)	1 (10.0%)	0 (0.0%)	1 (3.7%)
Not pregnant	3 (30.0%)	1 (10.0%)	1 (10.0%)	5 (50.0%)	8 (47.1%)	13 (48.1%)
Not applicable	2 (20.0%)	0 (0.0%)	1 (10.0%)	3 (30.0%)	9 (52.9%)	12 (44.4%)

### Demographic characteristics of IAV positive cases

**Fig. 2:**
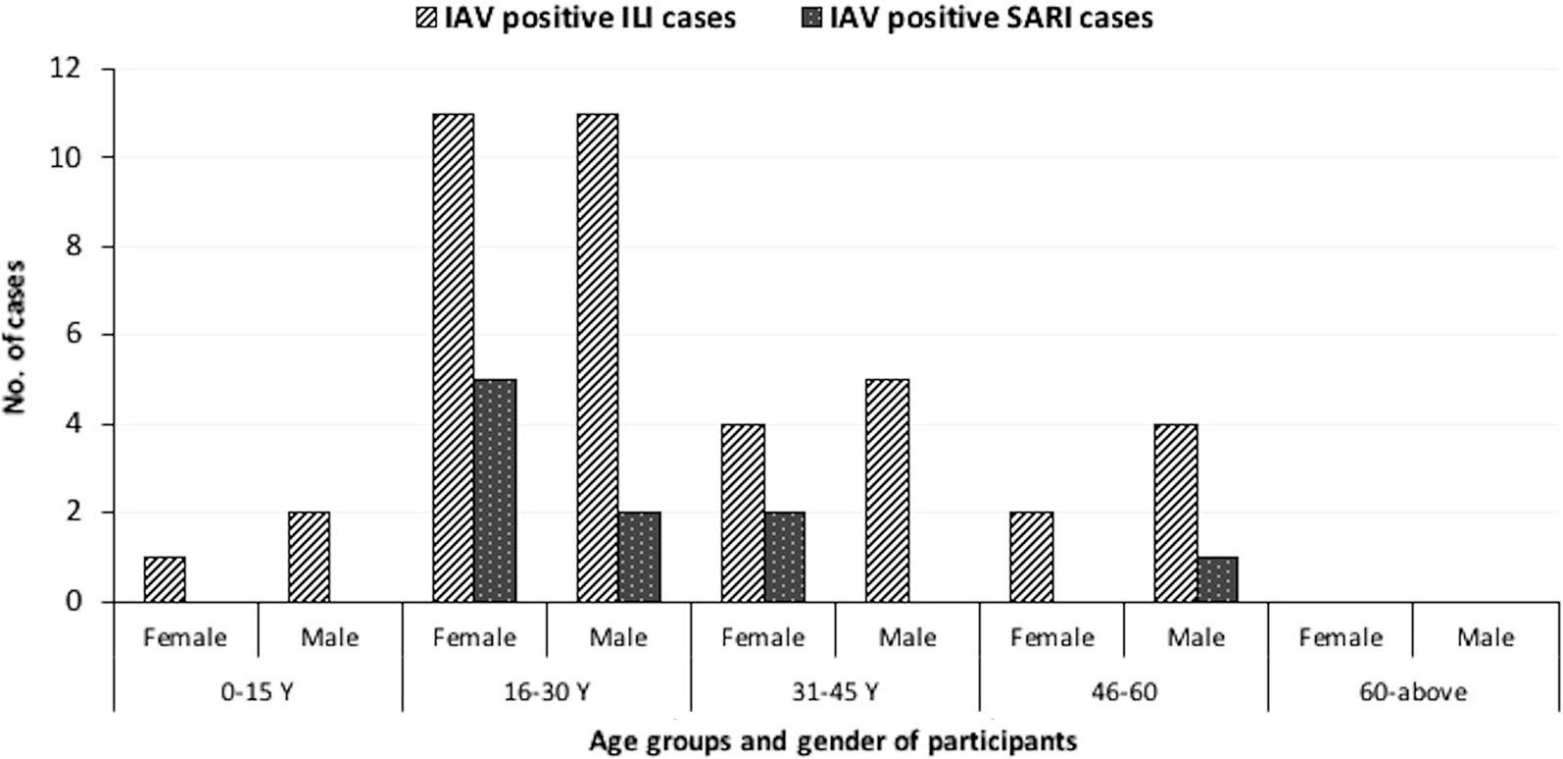
Distribution of positive IAV cases among ILI and SARI patients by age and gender

Among all enrolled individuals, 16% (50/311) were positive for IAV. Of these, 80% (n = 40) were from ILI and 20% (n = 10) were from SARI groups **(**Fig. 2). Among ILI cases, 14% (40/284) were IAV positive while among SARI cases 27% (10/27) were IAV positive.

### Temporal patterns of respiratory illnesses and Influenza A

**Fig. 3:**
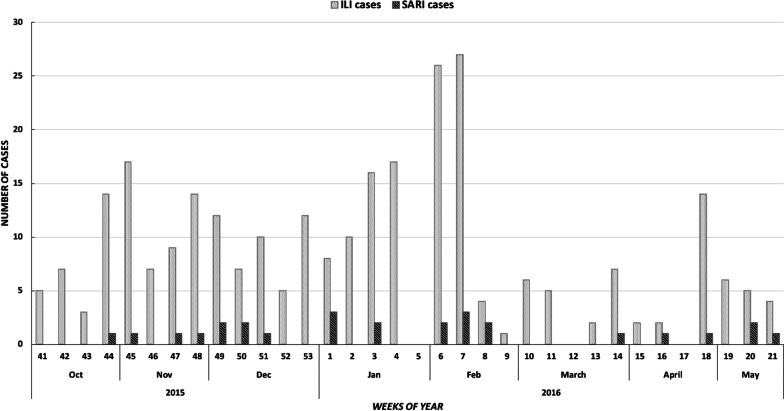
Temporal trend of ILI and SARI cases visiting sentinel hospital during October 2015 to May 2016 in Lahore, Pakistan

The proportion of respiratory illnesses (n = 4568) among all patients visiting the sentinel hospital during study period (n = 5,13,126) was 0.9% (95% Confidence Interval [CI]: 0.86–0.92). The proportion of enrolled ILI (284/311) was 91% (95% CI: 87.62–94.20) and enrolled SARI (27/311) was 8.68% (95% CI: 5.79–12.38). The maximum monthly number of ILI (20%) and SARI (26%) patients was seen in February (Fig. 3).

**Fig. 4:**
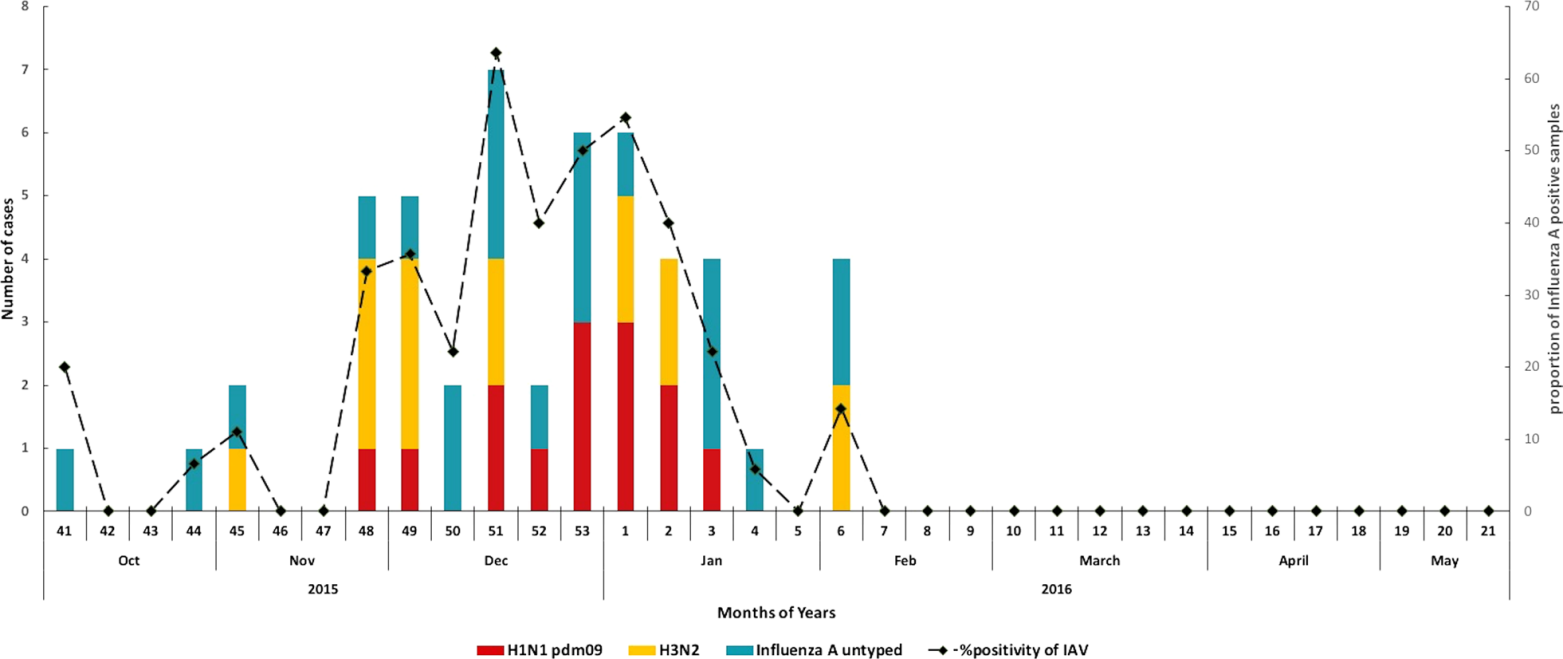
Number and percentage positivity of influenza-positive samples by duration of study (weeks, month) in Lahore, Pakistan

The proportion of IAV positive among all enrolled was 16% (50/311; 95% CI: 12.17–20.64). Among the 50 IAV positive cases, virus subtyping detected 14 (28%) as H1N1pdm09 and 15 (30%) as seasonal H3N2; the remaining 21 (42%) were untyped (Fig. 4). The detection of IAV was limited to winter months (December–February). The highest proportion (42%) of positive IAV cases was recorded during December 2015. (Fig. 4).

Among the ILI and SARI cases, 22.5% and 50% were H1N1pdm09 positive, 32.5% and 20% were seasonal H3N2 positive and 45% and 30% were untyped IAV positive respectively.

### Characteristics of IAV positive ILI cases

Among ILI patients, the number of IAV positives was highest among the 16–30 years (55%, 22/40). Among IAV positive ILI cases, 55% were male and 45% were female. No pregnant ILI females (n = 7) were IAV positive (Table 1).

### Characteristics of IAV positive SARI cases

Among SARI cases, the number of IAV positives was highest in 16–30 age group (70%, 7/10). Among IAV positive SARI cases 70% were female and 30% were male (Table 2). Both SARI pregnant females were IAV positive.

### Clinical characteristics and co-morbidities

**Fig. 5:**
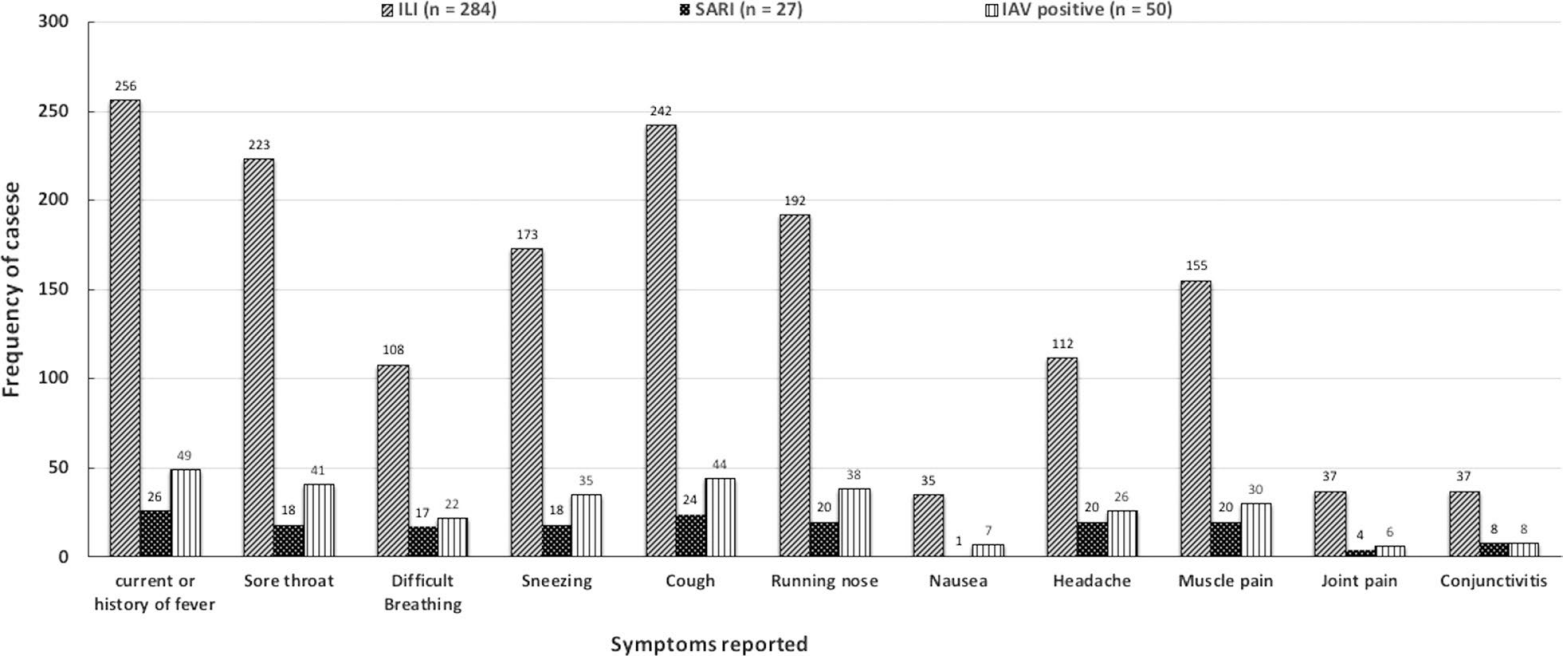
Distribution of clinical symptoms in ILI and SARI and IAV positive cases

Ongoing or history of fever was the most common symptom reported in the ILI (91%) and SARI (96%) groups. Cough (85%) and Sore throat (78.5%) were the other common clinical symptoms reported by ILI cases. Cough (89%), sore throat (67%), running nose (74%), headache (74%) and muscle pain (74%) were frequently reported symptoms by SARI cases (Fig. 5, Table 3).

**Table 3:** Distribution of influenza like illness (ILI) and severe acute respiratory illness (SARI) according to clinical symptoms of influenza and comorbidities during hospital based active surveillance

	Response	ILI (n = 284)		SARI (n = 27)	
		IAV positive (n = 40)	IAV negative (n = 244)	IAV positive (n = 10)	IAV negative (n = 17)
*Characteristics*					
Current or history of fever	No	1 (2.5%)	27 (11.1%)	1 (10%)	1 (5.9%)
	Yes	39 (97.5%)	217 (88.9%)	9 (90%)	16 (94%)
Sore throat	No	8 (20%)	53 (21.7%)	1 (10%)	8 (47.1%)
	Yes	32 (80%)	191 (78.3%)	9 (90%)	9 (52.9%)
Difficult breathing	No	25 (62.5%)	151 (61.9%)	3 (30%)	7 (41.2%)
	Yes	15 (37.5%)	93 (38.1%)	7 (70%)	10 (58.8%)
Sneezing	No	13 (32.5%)	98 (40.2%)	2 (20%)	8 (47.1%)
	Yes	27 (67.5%)	146 (59.8%)	8 (80%)	9 (52.9%)
Cough	No	5 (12.5%)	37 (15.2%)	1 (10%)	1 (5.9%)
	Yes	35 (87.5%)	207 (84.8%)	9 (90%)	16 (94.1%)
Running nose	No	9 (22.5%)	83 (34%)	3 (30%)	5 (29.4%)
	Yes	31 (77.5%)	161 (66%)	7 (70%)	12 (70.6%)
Nausea	No	34 (85%)	215 (88%)	9 (90%)	17 (100%)
	Yes	6 (15%)	29 (12%)	1 (10%)	0 (0%)
Vomiting	No	36 (90%)	221 (90.6%)	6 (60%)	14 (82.4%)
	Yes	4 (10%)	23 (9.4%)	4 (40%)	3 (17.6%)
Diarrhea	No	38 (95%)	227 (93%)	10 (10%)	13 (76.5%)
	Yes	2 (5%)	17 (7%)	0 (0%)	4 (23.5%)
Headache	No	22 (55%)	150 (61.5%)	2 (20%)	6 (35.3%)
	Yes	18 (45%)	94 (38.5%)	8 (80%)	11 (64.7%)
Seizures	No	39 (97.5%)	239 (98%)	10 (10%)	16 (94.1%)
	Yes	1 (2.5%)	5 (2%)	0 (0%)	1 (5.9%)
Altered consciousness	No	40 (100%)	238 (97.5%)	10 (10%)	16 (94.1%)
	Yes	0 (0%)	6 (2.5%)	0 (0%)	1 (5.9%)
Muscle pain	No	19 (47.5%)	110 (45%)	1 (10%)	7 (41.2%)
	Yes	21 (52.5%)	134 (56%)	9 (90%)	10 (58.8%)
Joint pain	No	36 (90%)	211 (86.5%)	8 (80%)	15 (88.2%)
	Yes	4 (10%)	33 (13.5%)	2 (20%)	2 (11.8%)
Epistaxis	No	39 (97.5%)	223 (91.4%)	10 (10%)	16 (94.1%)
	Yes	1 (2.5%)	11 (4.6%)	0 (0%)	1 (5.9%)
Conjunctivitis	No	34 (85%)	213 (87.3%)	8 (80%)	11 (64.7%)
	Yes	6 (15%)	31 (12.7%)	2 (20%)	6 (35.3%)
*Co-morbidities*					
Diabetes	No	39 (97.5%)	223 (91.4%)	10 (10%)	15 (88.2%)
	Yes	1 (2.5%)	11 (4.6%)	0 (0%)	2 (11.8%)
Heart diseases	No	39 (97.5%)	239 (98%)	10 (10%)	17 (100%)
	Yes	1 (2.5%)	5 (2%)	0 (0%)	0 (0%)
Seizures	No	40 (100%)	243 (99.6%)	10 (10%)	16 (94.1%)
	Yes	0 (0%)	1 (0.4%)	0 (0%)	1 (5.9%)
Lung diseases	No	40 (100%)	243 (99.6%)	10 (10%)	17 (100%)
	Yes	0 (0%)	1 (0.4%)	0 (0%)	0 (0%)
Asthma	No	40 (100%)	238 (97.5%)	10 (10%)	16 (94.1%)
	Yes	0 (0%)	6 (2.5%)	0 (0%)	1 (5.9%)
Allergy	No	40 (100%)	239 (98%)	10 (10%)	17 (100%)
	Yes	0 (0%)	5 (2%)	0 (0%)	0 (0%)
Hepatitis B	No	40 (100%)	241 (98.8%)	10 (10%)	17 (100%)
	Yes	0 (0%)	3 (1.2%)	0 (0%)	0 (0%)
Hepatitis C	No	38 (95%)	231 (94.7%)	10 (10%)	15 (88.2%)
	Yes	2 (5%)	13 (5.3%)	0 (0%)	2 (11.8%)
Obesity	No	39 (97.5%)	235 (96.3%)	9 (90%)	16 (94.1%)
	Yes	1 (2.5%)	9 (3.7%)	1 (10%)	1 (5.9%)
Malnutrition	No	40 (100%)	243 (99.6%)	10 (10%)	17 (100%)
	Yes	0 (0%)	1 (0.4%)	0 (0%)	0 (0%)
Immune deficiency/ HIV	No	40 (100%)	244 (100%)	10 (10%)	17 (100%)
	Yes	0 (0%)	0 (0%)	0 (0%)	0 (0%)
Tuberculosis	No	37 (92.5%)	235 (96.3%)	10 (10%)	17 (100%)
	Yes	3 (7.5%)	9 (3.7%)	0 (0%)	0 (0%)
Multiple conditions	No	39 (97.5%)	234 (95.9%)	10 (10%)	17 (100%)
	Yes	1 (2.5%)	10 (4.1%)	0 (0%)	0 (0%)
Other diseases	No	37 (92.5%)	219 (89.8%)	9 (90%)	16 (94.1%)
	Yes	3 (7.5%)	25 (10.2%)	1 (10%)	1 (5.9%)

The most common ILI co-morbidities were hepatitis C (5.2%), tuberculosis (4.2%) and diabetes (4.2%), while the most common co-morbidities in SARI cases were diabetes (7.4%), hepatitis C (7.4%) and obesity (7.4%). In all IAV positive cases, the most common comorbidities were hepatitis C (4%), obesity (4%) and tuberculosis (6%) (Table 3).

The majority of the patients reported to the hospital on day three post symptoms onset (66/311) (interquartile range [IQR] = 2–6 days) with 14 of these IAV positive (14/66).

### Spatial trend of cases

**Fig. 6:**
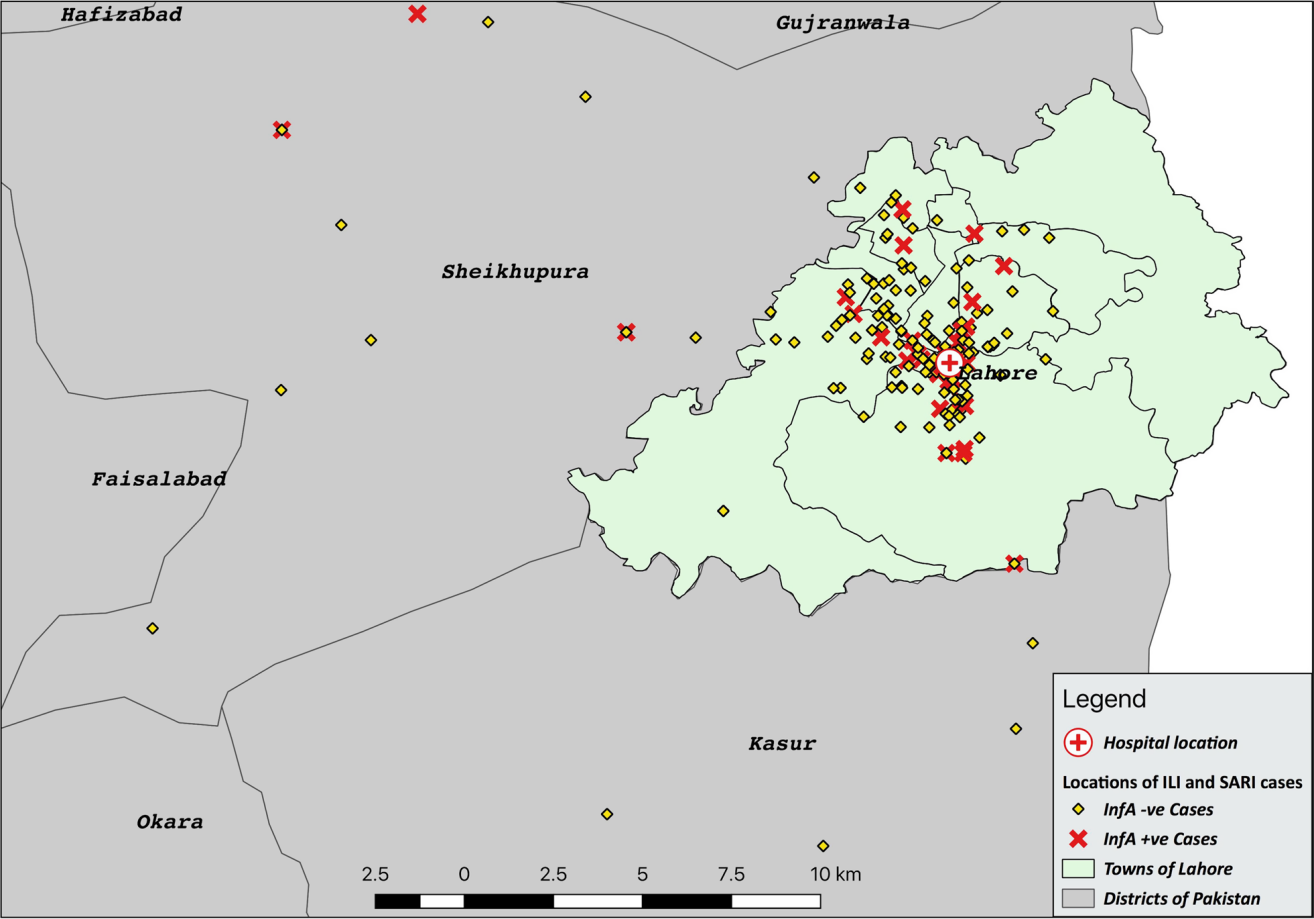
Spatial distribution of IAV cases during sentinel surveillance from Oct 2015 to May 2016

About 93.2% of the reported cases of ILI and SARI (290/311) lived in the Lahore district (Fig. 6); the remaining 21 were from other districts of Punjab province. Among the cases from Lahore district, 265 were ILI patients and 25 were SARI patients. Among IAV positive cases (n = 50), 47 were reported from Lahore district (94%).

## Discussion

In our study ILI and SARI cases reported to the sentinel site throughout the study period (October 2015–May 2016), however, a peak was seen in February 2016. Influenza virus was detected in our population during the wither months (December–February) with a distinct peak in December. Extended longitudinal studies are required to determine the reproducibility of this pattern and to identify interseasonal variations. Nevertheless, this temporal trend aligns with those presented in other studies from Pakistan [18, 28] and from other countries in temperate regions like the USA and China [29–32], suggesting similar impacts of the various factors that drive influenza seasonality including climate, host, and virus characteristics [32, 33]. In current study, influenza virus was not detected in samples from ILI & SARI patients, collected in week 46 and 47 (peak season for influenza), which might be attributed to small sample size (5–6 patients per day). Other respiratory viruses e.g. metapneumovirus, parainfluenza, respiratory syncytial virus, adeno and rhinovirus could be the etiology of ILI and SARI during those weeks [34, 35]. The overall proportion of IAV positive samples was 16% among enrolled patients, which is slightly lower than that previously reported (20%) in Pakistan [18] but within the range observed elsewhere (10–50%) [13, 31, 36]. The proportion of IAV among SARI cases was substantially higher than among ILI cases, a finding consistent with conclusions that SARI-based surveillance is a good data source for estimating influenza trends [31, 37]. Among the 50 IAV positive cases, 14 were subtyped as H1N1pdm09. H1N1pdm09 was the predominant IAV subtype circulating globally in the post-pandemic period (2011–2013), including in Pakistan [14, 33, 38]. The World Health Organization stated in 2010 that the pandemic virus will continue to circulate with seasonal viruses, causing occasional outbreaks [39] which it has [40, 41]. However, with the emergence of COVID-19 pandemic in December 2019, the circulation of influenza virus has dropped to historically low levels. One plausible reason for the low level of influenza virus circulation is the non-pharmaceutical interventions implemented for COVID-19 control; notably mask wearing and social distancing as the route of transmission of IAV and SARS-CoV-2 are similar i.e. aerosol [42].

Influenza viruses can infect individuals of all ages and the epidemiology of the resulting disease can be influenced by age groups [4, 24, 33, 36, 43, 44]. In our study, young adults (16–30 years) were found to have the highest percentage of IAV positivity among ILI (55%) and SARI (70%) patients. Previous influenza surveillance in Pakistan reported that individuals between 21–40 years and 41–60 years old, were most affected during the pre-pandemic, pandemic, and post-pandemic periods [45]. In other studies, relatively high proportions of IAV cases were seen among children aged 5–17 years [24, 43, 46]. In our study, the data about age of participants was not normally distributed, which might have influenced the analysis. In developing countries like Pakistan, young people (age 16–30 years) have the easiest access to healthcare facilities, while children and older people are dependent on young family member to take them. Contrary to our results, age disparity has been reported between ILI and SARI cases and influenza positivity in various studies [43, 47]. In addition to influenza viruses, other respiratory pathogens, e.g., parainfluenza viruses and respiratory syncytial virus contribute to respiratory illness in children and the elderly [48]. In the current study, the proportion of IAV positive cases was higher among male ILI patients (55%) than female ILI cases (45%), while in SARI patients, the proportion of IAV positive cases was higher among female (70%) than male (30%). Males and females have been shown to have variation in susceptibility to influenza infection, which might be partially explained by the fact that sex-specific endocrines can affect immune responses [49, 50]. Further studies are needed to collect scientific evidence on the combined effects of age and sex as biological variables influencing IAV infection and resulting disease severity.

In our study 99.4% of participants did not know about seasonal influenza vaccine and only one participant was vaccinated with seasonal influenza vaccine. The World Health Organization highly recommends influenza vaccination on a yearly basis, especially for children and the elderly people [51]. Various factors drive low uptake in Pakistan including the relatively high cost, customs and cultural practices and low literacy rates [52].

We also analyzed the clinical characteristics of ILI, SARI and IAV positive cases. Almost all ILI and SARI (> 90%) and IAV positive cases (98%) presented with ongoing or history of fever. The second most common symptom reported by ILI, SARI and IAV positive cases was cough. A similar study conducted in Europe reported fever, malaise and cough as the most common symptoms of influenza [38]. Many other studies have suggested that influenza could be maximally predicted in patients presenting with clinical symptoms of fever and cough [13, 53–56]. Our results endorse the revised influenza case definition by WHO published in 2011 and suggest fever and cough as the main clinical predictors for influenza among ILI and SARI cases [57].

We found that hepatitis C, tuberculosis, and obesity were the most common co-morbidities in IAV positive cases. Influenza infection causes hepatic decompensation in patient with chronic liver disease and may cause hepatitis itself by stimulating liver-damaging toxic metabolites and pro-inflammatory cytokines [58]. Similarly, obesity can be thought of as a form of immunosuppression resulting in more severe pathogenesis following infection [59]. Consistent with our findings, tuberculosis has been previously identified as a risk factor for mortality in IAV patients [60].

Most of the patients in our study arrived at the hospital within three days of onset of their symptoms, a timepoint when we also had maximum detection of IAV. Detection of IAV is more likely if samples are taken within 7 days of infection [61]. Systemic symptoms such as myalgia, headache, and fever are due to cytokine release from immune cells and appear rapidly after viral infection is detected by the immune system. Symptoms of rhinorrhea and nasal congestion are produced by inflammatory mediators (prostaglandins and bradykinin) and are slower to develop but longer lasting. Accurate symptomatic diagnosis of pandemic virus infections including influenza and coronavirus is essential for the initiation of public health interventions in the community but is an area that require much research [62]. However, relying on clinical diagnosis of influenza alone will not be sufficient, as symptoms caused by other pathogens can overlap considerably and diagnostic testing must be used to aid clinical judgment and help guide treatment decisions and vaccine development [63].

The major limitations of our study were that all age groups were not equally represented in the study population. Due to logistic and access issues, we only sampled 5–6 patients on each day which might have introduced selection bias in our findings towards severely ill patients or those that lived closer to the hospital and arrived earlier. Hence, our results are probably an underestimation of the proportion of people with ILI or SARI and the proportion of those that are IAV positive. Another limitation is that we only analyzed samples for IAV and these data may not be directly generalizable to influenza B viruses. One sentinel hospital was included for data collection for a period of only 8 months, a factor that also limit the confidence in our representative estimates. Similarly, our surveillance included a small number of pregnant women (n = 9) and children (n = 41), who attended outpatient clinics, and our data on these groups may not reflect the population in pediatric or obstetric wards. As our surveillance was hospital-based, the denominator of our catchment area’s population was unavailable, therefore, cumulative incidence of influenza could not be estimated.

## Conclusions

In the current study, we conclude that ILI, SARI and IAV positive cases have distinct characteristics and temporal trends. These trends may prove useful to health policy makers to initiate public health interventions at appropriate times among high-risk groups. We highly recommend inclusion of sampling from both ILI and SARI patients in routine surveillance as suggested by WHO. Influenza vaccination should be included in the expanded program on immunization (EPI) of Pakistan, which aims to decrease childhood morbidity and mortality due to vaccine preventable diseases.

## Supplementary Information

**Additional file 1.** Questionnaire for data collection.

## Data Availability

The data gathered and generated during the current study are available from the corresponding author (Mamoona Chaudhry) on reasonable request.

## Abbreviations

ILI: Influenza like illness
SARI: Severe acute respiratory syndrome
IAV: Influenza A virus
IQR: Interquartile range
LGH: Lahore General Hospital
rRT-PCR: Real-time reverse transcriptase polymerase chain reaction
